# Characteristics and Prognoses of Long-Term Home Care Patients

**DOI:** 10.1093/geroni/igab046.2341

**Published:** 2021-12-17

**Authors:** Takashi Yamanaka, Maiko Mizuki, Kiwami Kidana, Ryonosuke Yamaga

**Affiliations:** 1 The University of Tokyo, Bunkyo-ku, Tokyo, Japan; 2 Tsubasa Home Care Clinic, Funabashi-city, Chiba, Japan

## Abstract

With demographic aging, many older adults require home medical care. Although home-based primary care is promoted in the United States and Japan, there is insufficient evidence about it. We aimed to study the characteristics and prognoses of long-term home care patients. We prospectively registered 151 patients, estimated to receive physician home visits for more than six months, in a clinic in Chiba, Japan, in 2020. The mean (±SD) age was 83.9±10.0 years and ranged from 31 to 102 years. Most patients were men (60.3%) and aged 65 years or above (95.3%). We investigated clinical information, the Edmonton Symptom Assessment System Revised Japanese version (ESAS-r-J), Dementia Assessment Sheet in Community-based Integrated Care System 21 items (DASC-21), EuroQOL 5 dimensions 5-level (EQ-5D-5L) every six months, and the incidence of hospital admission, death, and patient transportation by ambulance. The most frequent diagnoses were dementia (31.1%), bone and articular diseases (17.2%), cerebrovascular diseases (11.9%), organ failure (9.3%), and neurological diseases (9.3%). Most patients (78.2%) showed more than 30 points on the DASC-21, suggesting cognitive impairment. Worse wellbeing, drowsiness, tiredness, anxiety, depression, and pain were the most prevalent symptoms. EQ-5D-5L index values were distributed around–0-0.2 and 0.4-0.7. During the first three months of physician home visits, 21.9% of patients had hospital admissions, 12.5% of them died, and 11.7% required hospital transportation by an ambulance. In this study, most long-term home care patients suffered from cognitive impairment. In addition to receiving care for daily life, these patients require intensive medical management.

